# Assessment of knowledge, attitude and practices and the analysis of risk factors regarding schistosomiasis among fishermen and boatmen in the Dongting Lake Basin, the People’s Republic of China

**DOI:** 10.1186/s13071-020-04157-4

**Published:** 2020-06-01

**Authors:** Zhou Guan, Si-Min Dai, Jie Zhou, Xiao-Bing Ren, Zhi-Qiang Qin, Yin-Long Li, Shan Lv, Shi-Zhu Li, Xiao-Nong Zhou, Jing Xu

**Affiliations:** 1 National Institute of Parasitic Diseases, Chinese Center for Disease Control and Prevention, Shanghai, People’s Republic of China; 2 Key Laboratory of Parasite and Vector Biology, National Health Commission, Shanghai, People’s Republic of China; 3 WHO Collaborating Centre for Tropical Diseases, Shanghai, People’s Republic of China; 4 Chinese Center for Tropical Diseases Research, Shanghai, People’s Republic of China; 5 Hunan Institute of Schistosomiasis Control, Yueyang, People’s Republic of China; 6 Yueyang County Office for Preventive and Control on Schistosomiasis, Yueyang, People’s Republic of China

**Keywords:** Schistosomiasis, Knowledge, Attitude, Practice, Prevalence, Influencing factors, Logistic regression

## Abstract

**Background:**

Fishermen and boatmen are a population at-risk for contracting schistosomiasis due to their high frequency of water contact in endemic areas of schistosomiasis in the People’s Republic of China (P. R. China). To develop specific interventions towards this population, the present study was designed to assess the knowledge, attitudes and practices (KAPs) towards schistosomiasis of fishermen and boatmen, and to identify the risk factors associated with schistosome infection using a molecular technique in a selected area of Hunan Province in P. R. China.

**Methods:**

A cross sectional survey was conducted in the Dongting Lake Basin of Yueyang County, Hunan Province. A total of 601 fishermen and boatmen were interviewed between October and November 2017. Information regarding sociodemographic details and KAPs towards schistosomiasis were collected using a standardized questionnaire. Fecal samples of participants were collected and tested by polymerase chain reaction (PCR). Logistic regression analysis was conducted to explore the risk factors related to the positive results of PCR.

**Results:**

Of the 601 respondents, over 90% knew schistosomiasis and how the disease was contracted, the intermediate host of schistosomes and preventive methods. The majority of respondents had a positive attitude towards schistosomiasis prevention. However, only 6.66% (40/601) of respondents had installed a latrine on their boats, while 32.61% (196/601) of respondents defecated in the public toilets on shore. In addition, only 4.99% (30/601) respondents protected themselves while exposed to freshwater. The prevalence of schistosomiasis, as determined by PCR, among fishermen and boatmen in Yueyang County was 13.81% (83/601). Age, years of performing the current job, number of times receiving treatment, and whether they were treated in past three years were the main influencing factors of PCR results among this population.

**Conclusions:**

Fishermen and boatmen are still at high risk of infection in P. R. China and gaps exist in KAPs towards schistosomiasis in this population group. Chemotherapy, and health education encouraging behavior change in combination with other integrated approaches to decrease the transmission risk in environments should be improved.
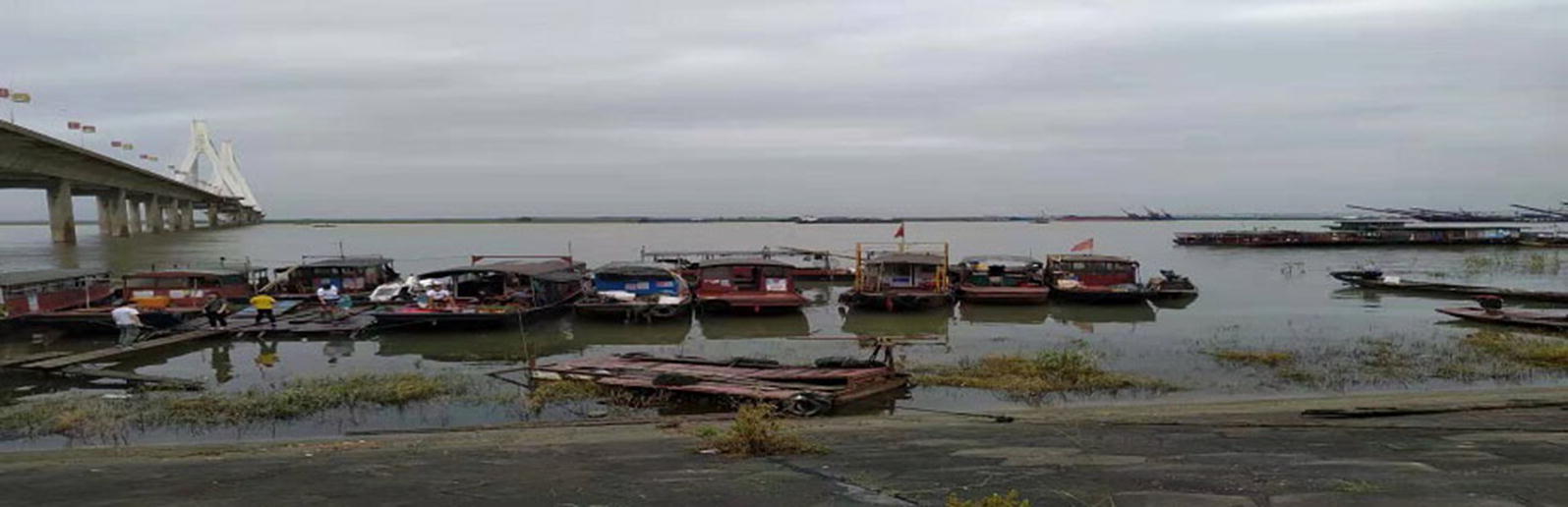

## Background

Schistosomiasis has been considered to be one of neglected tropical diseases of public health importance in tropical and subtropical areas of the world [1–3]. It is endemic in 78 countries and territories globally with 250 million people infected [4]. The People’s Republic of China (P. R. China) used to have the world’s highest disease burden of schistosomiasis caused by *Schistosoma japonicum.* National surveys conducted in the 1950s estimated that P. R. China had 11.60 million human cases and one million infected cattle [5]. Schistosomiasis is acquired when people or domestic animals contact freshwater contaminated with free swimming cercariae of schistosomes.

During the past seven decades, great efforts have been made to control schistosomiasis through consecutive and vertical control programs by the Chinese government. This is especially true over the past decade when preventing schistosomiasis was ranked as a high priority among four important infectious diseases nationally, including HIV/AIDS, tuberculosis and hepatitis B [6]. During that time, an integrated control strategy was also implemented for schistosomiasis according to the medium- and long-term national control plan (2004–2015) [7–9]. The prevalence and intensity of infections with *S. japonicum* in humans has been reduced substantially, and the number of endemic provinces has decreased from twelve to seven [10, 11]. Currently schistosomiasis is mainly endemic in areas surrounding the Dongting Lake, Poyang Lake and beaches along the Yangtze River [12–15], with fishermen, boatmen and farmers being groups at high risk of infection and suffering the highest disease burden [16].

In spite of the great success in schistosomiasis control in P. R. China as a result of these programmes, there are still enormous difficulties and challenges surrounding transmission interruption and the elimination of schistosomiasis [17]. Potential risk factors for the resurgence of schistosomiasis still exist and the prevalence of schistosomiasis can rapidly rebound in the lake and marshland region due to its unique environment [18]. Tools used to determine the infection status or prevalence of schistosomiasis in humans are still heavily reliant on traditional methods, such as Kato-Katz, which presents the increasingly obvious disadvantage of poor sensitivity, especially in low endemic situations of mild infection [19, 20]. The false negative result could eventually contribute to inappropriate decisions or policies made by the government. According to the national surveillance data, floating populations, in particular, fishermen and boatmen, are becoming increasingly significant in schistosomiasis transmission [21]. Reinfection with *S. japonicum* among fishermen and boatmen is still a key issue impeding the transmission interruption or elimination of schistosomiasis in the lake and marshland regions in P. R. China [16].

Adequate knowledge, positive attitudes and the correct preventive practices of populations towards schistosomiasis prevention and control in endemic regions can provide an effective sustainable environment for the success and sustainability of schistosomiasis control [22–24]. Although health education is an important approach for schistosomiasis intervention, information on the current infection status and KAPs on schistosomiasis among fishermen and boatmen remains unclear. This study aimed at assessing the KAPs towards schistosomiasis among fishermen and boatmen in selected areas within Hunan Province (P. R. China). Infectious status and its influencing factors were also identified in this population.

## Methods

### Study design

This study was a cross-sectional survey that assessed the knowledge, attitude and practices (KAPs) on schistosomiasis, evaluated the prevalence of schistosomiasis and its risk factors among fishermen and boatmen in the study area.

### Study area and population

**Fig. 1:**
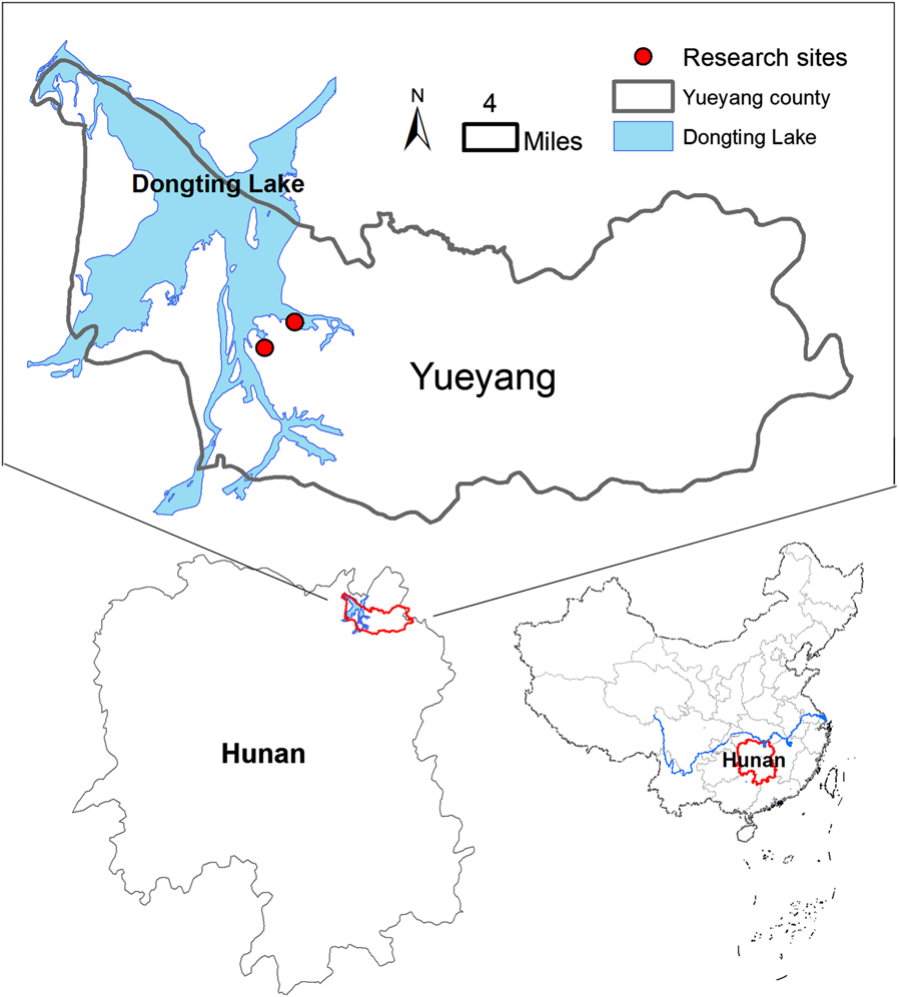
Location of study area in Yueyang County, Hunan Province

The study was conducted between October and November 2017 in Yueyang county, Hunan Province, which is located on the eastern bank of the Dongting Lake in southern China (Fig. 1). Two villages, Lujiao and Matang, in this county were sampled randomly from fishing villages with 3–5% prevalence of schistosomiasis determined by stool examinations in 2016. As typical lake and marshland endemic areas of schistosomiasis, these villages had numerous fishermen and boatmen at high risk of schistosome infection. Fishermen and boatmen in this region commonly live or work on ships for at least four months per year, and their economic income mainly comes from fishing and goods transportation. The respondents in our study comprised of either professional or non-professional fishermen and boatmen. All participants involved had not received any anti-schistosome treatment within a period of six months before the study. The interviews were conducted when their ships berthed at Lujiao and Matang in Yueyang County, Hunan Province, P. R. China.

### Questionnaire survey

A pretested semi-structured questionnaire was developed after performing a thorough literature review of comparable studies [22, 25–28], to assess the KAPs of participants towards schistosomiasis prevention. This survey was conducted by well-trained interviewers who received training by professionals from the National Institute of Parasitic Diseases, and was monitored by quality control supervisors who were assigned to each group before the interviews. The questionnaire consisted of four main sections (Additional file 1: Table S1). The first section covered sociodemographic information such as age, gender, education, family income, years of practice, history of receiving treatment against schistosomiasis. The second section covered participants’ knowledge of schistosomiasis, including the transmission season, susceptible population, definitive host, transmission mode, intermediate host, clinical manifestations, impacts on children and females, preventive methods, and anti-schistosome medication. The third section covered participants’ attitudes towards schistosomiasis prevention. The last section interviewed participants’ practices on schistosomiasis prevention. The full questionnaire is provided in Additional file 1: Table S1.

### Sampling collection

One fecal sample from each participant was collected using a specific container, and coded by a unique identifier. The samples were then transferred to a laboratory in the local schistosomiasis control station. Two grams of feces from each sample was filtered using a nylon membrane and placed in a cryopreserved tube at − 20 °C for one month prior to DNA extraction.

### Laboratory study

Total DNA of stool samples was extracted using the QIAamp DNA Stool Mini kit (Qiagen, Hilden, Germany) according to the manufacturer’s protocols. Genomic DNA extracted was subjected to PCR using 18S-FW (5′-TTC CGA TAA CGA ACG AGA C-3′) and 18S-RV (5′-AGC GAT AAA GCC ACT ACA AC-3′) specific primers amplifying a 469 bp region of the *S. japonicum 18S* rRNA gene [29]. Each PCR reaction contained 2 μl of template DNA, 25 μl of dNTPs mix (Tiangen Biotech Co., Ltd, Beijing, China), 1 μl of upstream and downstream primers, and 21 μl of sterile double-distilled water. The PCR conditions were as follows: 5 min at 94 °C (initial denaturation), 30 cycles of 20 s at 94 °C, 30 s at 56 °C, 40 s at 72 °C, and a final extension step at 72 °C for 5 min. A negative control (sterile double-distilled water) and positive control (worm body tissue DNA) were set up in each batch of experiments. The PCR products were separated on 1.5% agarose gels. All positive PCR amplification products for the *18S* RNA gene were sequenced, and compared with sequences published on GenBank using BLAST to check the consistency with the targeted *18S* rDNA sequence.

### Data management and statistical analysis

Data collected were entered into a database using EpiData Version 3.0 (https://www.epidata.dk) and then analyzed using SAS Version 9.4 (Statistical Analysis System, RTI, Cary, North Carolina, USA). The analysis consisted of three parts. First, descriptive statistics were used for the demographic characteristics of the respondents. Secondly, percentages of variables related to schistosomiasis control knowledge, attitude, and practices were calculated to provide information on the KAPs of *S. japonicum* among the respondents. Finally, the *Schistosoma* nucleic acid positive rate was calculated according to the results of PCR amplification and electrophoresis. Risk factors related to the positive results of PCR were analyzed by univariate logistic regression followed by stepwise regression and multivariate logistic regression model. *P-*values less than 0.05 were considered to be statistically significant.

## Results

### Sociodemographic characteristics

A total of 753 fishermen and boatmen were enrolled in this cross-sectional study. However, 122 people provided none or insufficient amounts of stool to prepare for PCR and 30 individuals had no or incomplete questionnaire data. Therefore, 601 individuals who completed the questionnaire and provided adequate stool samples were included in the final analysis. The sociodemographic characteristics of the respondents are presented in Table 1. The number of males was 345 (57.40%) and females was 256 (42.60%). The mean age of respondents was 50.04 years-old (standard deviation, SD = 10.97). Of the 601 respondents, 59.90% (360/601) were non-professional fishermen and boatmen, while 38.27% (230/601) and 1.83% (11/601) were professional boatmen and fishermen respectively. The majority (63.39%, 381/601) had been in their current job for 10–29 years, followed by those who have had their job for more than 30 years (128/601, 21.30%). Over 95% of respondents (576/601) had received treatment against schistosomiasis at least once. The majority of participants (72.05%, 433/601) were from families having an annual household income less than 10,000 Chinese Yuan (about 1511 USD).

**Table 1:** Major sociodemographic characteristics of the respondents attending KAPs survey and provided stool samples

Variables	Category	Frequency	Percentage (%)
Gender	Male	345	57.40
	Female	256	42.60
Occupation	Professional fisherman	11	1.83
	Professional boatman	230	38.27
	Non-professional fisherman and boatman	360	59.90
Age group (years)	< 30	21	3.49
	30–39	54	8.99
	40–49	228	37.94
	50–59	184	30.62
	≥ 60	114	18.97
Education	Below primary school	57	9.48
	Primary school	167	27.79
	Junior middle school	324	53.91
	High school and higher education	53	8.82

### Participants’ KAPs towards schistosomiasis

#### Knowledge about schistosomiasis

Of the 601 respondents, 74.88% (450/601) knew that the susceptible season for infection with *S. japonicum* is from April to October and 72.05% (433/601) knew that the general population is susceptible to infection with schistosomes. The majority (95.67%, 575/601) of respondents mentioned snails as the intermediate host for *S. japonicum* while 75.04% (451/601) of them knew the definitive host as humans and livestock. Of those surveyed, 97.00% (583/601) mentioned contact with *S. japonicum*-infested water as the way to become infected. In terms of participants’ knowledge on symptoms and effects of schistosomiasis, 86.69% (521/601) of respondents mentioned fever and diarrhea as the main symptoms of schistosomiasis, while 58.40% (351/601) said that schistosomiasis could have a severe impact on females, and 62.73% (377/601) indicated that schistosomiasis could limit children’s physical growth. With respect to preventive activities and treatment for schistosomiasis, 96.17% (578/601) of respondents mentioned avoiding contact with *S. japonicum*-infested water as an effective preventive measure. Only 47.92% (288/601) of respondents mentioned praziquantel as an anti-schistosome drug (Table 2).

**Table 2:** Knowledge of schistosomiasis among the respondents

Knowledge regarding schistosomiasis	Response	Frequency	Percentage (%)
Transmission season (CA: April to October)	Correct	450	74.88
	Incorrect	151	25.12
Susceptible population (CA: Everyone)	Correct	433	72.05
	Incorrect	168	27.95
Definitive hosts (CA: Humans and livestock)	Correct	451	75.04
	Incorrect	150	24.96
Intermediate hosts (CA: *Oncomelania*)	Correct	575	95.67
	Incorrect	26	4.33
Cause of infection (CA: Contact infected water)	Correct	583	97.00
	Incorrect	18	3.00
Main symptoms (CA: Fever, diarrhea)	Correct	521	86.69
	Incorrect	80	13.31
Effects on children (CA: Stagnant physical growth, etc.)	Correct	377	62.73
	Incorrect	224	37.27
Effects on women (CA: Infertility, etc.)	Correct	351	58.40
	Incorrect	250	41.60
Preventive measures (CA: Do not have contact with infected water)	Correct	578	96.17
	Incorrect	23	3.83
Medicine used for treatment (CA: Praziquantel)	Correct	288	47.92
	Incorrect	313	52.08

*Abbreviation*: CA, correct answer

#### Attitude to schistosomiasis prevention and control

Most respondents (95.34%, 573/601) indicated that they were willing to install toilet facilities on their boats. Of the 28 respondents who refused to install toilet facilities, 57.14% complained that fishing boats were too small to accommodate any toilet facility. With regard to visiting onshore public toilets, 96.17% (578/601) reported that they would like to use onshore public toilets, while 72.7% of respondents who were reluctant to use onshore public toilets gave the reason that it was inconvenient to go ashore to find toilets over such a long distance when they were working in the middle of the lake. The majority of respondents (98.50%, 592/601) were willing to accept examination for schistosomiasis and 98.50% (592/601) of the respondents expressed willingness to take medicine regularly following doctors’ prescriptions if diagnosed as positive. Of those surveyed, 89.85% (540/601) believed this disease could be prevented, while 71.88% (432/601) understood schistosomiasis could be cured (Table 3).

**Table 3:** Attitude and practices toward schistosomiasis prevention and control among the respondents

Attitude and practices toward schistosomiasis	Category	Frequency	Percentage (%)
Willingness to install toilet facilities	Yes	573	95.34
	No	28	4.66
Willingness to use onshore public toilets	Yes	578	96.17
	No	23	3.83
Willingness to accept examination	Yes	592	98.50
	No	9	1.50
Willingness to take medicine	Yes	592	98.50
	No	9	1.50
Belief that schistosomiasis could be prevented	Yes	540	89.85
	No	61	10.15
Belief that schistosomiasis could be cured	Yes	432	71.88
	No	169	28.12
Install and use toilet facilities	Yes	40	6.66
	No	561	93.34
Defecate in public toilets onshore	Yes	196	32.61
	No	405	67.39
Protection when contacted water	Yes	30	4.99
	No	571	95.01

#### Practices towards schistosomiasis

Of the 601 respondents, only 6.66% installed and used fecal containers in the correct way. In terms of protective behaviors when contacting *S. japonicum-*infested water, only 4.99% (30/601) of the respondents reported always wearing rubber shoes, gloves, protective clothing or using ointment on their skin. The main reason given by respondents who did not protect themselves when contacting freshwater in the Dongting Lake was that it was a bother (83.98%, 471/561). Only 32.61% (196/601) of the participants reported always defecating in onshore public toilets (Table 3).

### *Schistosoma japonicum* infection status and related risk factors

**Fig. 2:**
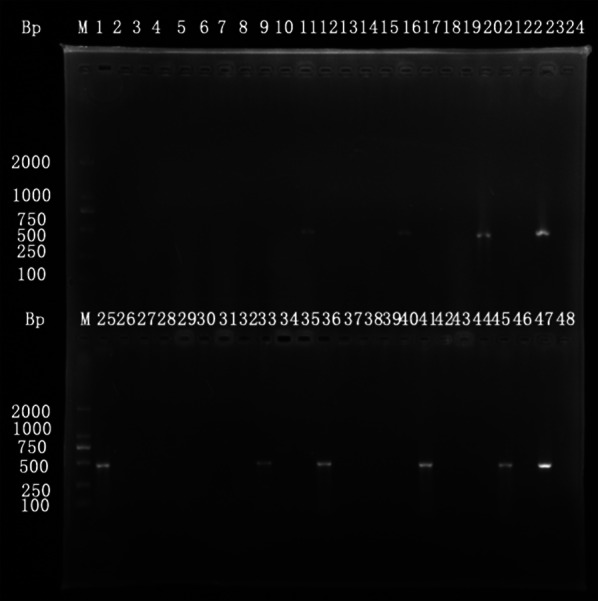
Gel electrophoresis of representative PCR results of the respondents

Among 601 participants who provided adequate stool samples, 13.81% (83/601) were detected as positive by PCR. The PCR amplification products of all positive samples were sequenced and the results showed that the DNA fragment was 469 bp, which was consistent with the target DNA both in length and sequence (Fig. 2). The positive rate of *Schistosoma* DNA in males (14.78%) was higher than that of females (12.50%), but not statistically different (*χ*^2^ = 0.643, *df* = 1, *P* = 0.423). The subgroup of participants who were aged less than 30 years-old, performing their current job for more than 30 years, never been treated for schistosomiasis, or not received treatment during 2015–2017, presented the highest PCR positive rates of 38.10% (8/21), 21.09% (27/128) and 32.00% (8/25), respectively, when conducting strata analysis (Table 4).

**Table 4:** Multiple logistic regression analysis of variables associated with *S. japonica* among the respondents

Risk factors	Positive rate (%) determined by PCR (No. positives/No. examined)	OR (95% CI)	*P*-value
Age group (years)			
< 30	38.10 (8/21)	1	
30–39	27.78 (15/54)	0.318 (0.075–1.354)	0.1212
40–49	15.35 (35/228)	0.163 (0.041–0.644)	0.0096*
50–59	10.33 (19/203)	0.058 (0.014–0.239)	< 0.0001*
≥ 60	5.26 (6/114)	0.012 (0.002–0.065)	< 0.0001*
Years of doing current job			
< 10	7.61 (7/92)	1	
10–29	12.86 (49/381)	4.162 (1.461–11.856)	0.0076*
≥ 30	21.09 (27/128)	18.684 (5.430–64.295)	< 0.0001*
Treatment times			
0	32.00 (8/25)	1	
1–5	17.13 (37/216)	0.265 (0.064–1.095)	0.0666
6–9	12.26 (26/212)	0.156 (0.035–0.689)	0.0142*
≥ 10	8.11 (12/148)	0.100 (0.021–0.476)	0.0038*
Treatment in 2015–2017			
No	18.18 (40/220)	1	
Yes	11.29 (43/381)	0.479 (0.246–0.930)	0.0298*

*Significant association (*P* < 0.05)

*Abbreviations*: OR, odds ratio; CI, confidence interval

Based on univariate logistic regression analysis (Additional file 2: Table S2), nine independent variables were included in the multivariate logistic regression, including age, occupation, economic conditions, years of doing current job, infection history, diagnostic method, disease category, times received treatment, and whether received anti-schistosome treatment from 2015–2017.

The results of multivariate logistic regression analysis indicated that respondents aged 40–49, 50–59 and ≥ 60 years-old had significantly lower odds of being positive than younger groups (< 30, 30–39), as determined by PCR (OR_40–49_ = 0.163, 95% CI: 0.041–0.644; OR_50–59_ = 0.058, 95% CI: 0.014–0.239; OR_≥60_ = 0.012, 95% CI: 0.002–0.065). The fishermen and boatmen who had performed their current job for 10–29 years and over 30 years were more likely to be PCR positive (OR_10–29_ = 4.162, 95% CI: 1.461–11.856 and OR_≥30_ = 18.684, 95% CI: 5.430–64.295, respectively) (Table 4). In addition, the respondents who received treatment 6 to 10 times, or more than 10 times showed lower odds of presenting positive PCR results (OR_6–9_ = 0.156, 95% CI: 0.035–0.689) and OR_≥10_ = 0.100, 95% CI: 0.021–0.476), compared to those never receiving treatment for schistosomiasis. Respondents who received treatment from 2015 to 2017 were more likely to have negative PCR results (OR = 0.479, 95% CI: 0.246–0.930) (Table 4).

## Discussion

The study showed that the overall awareness of schistosomiasis control knowledge among respondents was satisfactory, which was attributed to the persistent health education conducted over the years [30–32]. Despite sufficient knowledge and good attitudes towards schistosomiasis prevention, the behavioral practices of fishermen and boatmen were quite unsatisfactory. People preferred to receive examinations and treatment, rather than to prevent disease altogether. Only 6.66% of respondents had installed toilet facilities on their boats and only 32.61% of respondents defecated in the onshore public toilets. There were many reasons for such findings given by the interviewed fishermen and boatmen, including the boat size and stools could overflow from containers because of boat instability, or that fecal odors could make such containers intolerable on board. Of the respondents, only 4.99% protected themselves when contacting freshwater, and the main concern of such respondents was that it was troublesome and inconvenient to wear protection apparatus when fishing or working on the water. Adequate knowledge and positive attitudes did not convert to effective behavior changes, mainly due to the strong economic drive of their work, which agreed with previous studies [22, 33].

The gaps observed between knowledge, attitude and practices indicated that effective methods and enormous efforts should be made to better address the problem of transmission caused by behavior approaches. First, appropriate health promotion activities should be provided to this high-risk population. Secondly, existing interventions should be improved or modified to be more accessible and attractive to fishermen and boatmen. Finally, integrated approaches to eliminate the transmission risk of schistosomiasis in freshwater areas should be further explored and strengthened [14]. The prohibition of fishing activities is going to be implemented along the Yangtze River, which can assist in handling the current situation. An effective monitoring system needs to be established and run efficiently to guarantee this policy.

In P. R. China, a two-step detection pattern with serological screening first, followed by stool examinations for serological positives is used for the field diagnosis of schistosomiasis in China [16]. This method presented great advantages in earlier programmes because of its enormous capacity for large-scale screening and examination. However, with the persistent implementation of integrated control strategies for schistosomiasis, the prevalence and intensity of schistosomiasis in China has reached a historically low level [20, 34]. The infected fishermen and boatmen can be easily misdiagnosed by traditional methods, such as the Kato-Katz method or the miracidia hatching technique, and therefore, are not targeted for medication, which brings great challenge to the elimination of schistosomiasis in China [35, 36].

As a classic DNA amplification technique, PCR has many advantages over stool examinations, such as higher sensitivity and specificity, the low cost of reagents, and the availability of universal equipment in laboratories, which will facilitate schistosomiasis detection in the future [37]. It was reported that only 2.16 eggs per gram of feces were required to detect schistosomiasis by PCR [37]. This study found a high burden of schistosomiasis in fishermen and boatmen in Yueyang County using PCR (13.81%), which is higher than the average prevalence obtained from the national surveillance system [16, 22, 38]. The following considerations are therefore initiated for discussion. First, the study population generally spend most of their lives onboard and are in frequent contact with *S. japonicum-*infested water, which is thought to be the main reason for their vulnerability to infection and re-infection by schistosomes [36, 39]. Secondly, interventions against schistosomiasis were difficult to cover all fishermen and boatmen due to their high mobility [25, 40]. Although treatment should be delivered to fishermen and boatmen with high risk of schistosome infection according to the local treatment strategy, 4.16% of respondents had never received treatment and 36.61% had only received treatment two years before our study. Thirdly, our study used a DNA-based detection method rather than traditional methods. The PCR technique used in this study had an excellent sensitivity as it could detect 10 pg/μl of schistosome genomic DNA (based on internal assessments), as well as great specificity reflected by DNA sequencing of the PCR products. Therefore, PCR could facilitate the diagnosis and treatment of individuals with low-level infections and assist in the elimination of schistosomiasis in P. R. China.

Logistic regression analyses showed that four variables, i.e. age, years of performing the current job, times received treatments in history, and whether received treatments in the past three years, were significantly associated with the PCR results. Younger fishermen and boatmen are more likely to be infected by schistosomes, which is similar to results of previous research [25]. As the main labor forces of their families, young fishermen and boatmen are more likely to be involved in fishing or household chores, which means higher frequency and greater level of exposure to *S. japonicum*-infested water, therefore, increasing the likelihood for infection. In addition, there are also studies showing that aged fishermen and boatmen with long-term exposure to water can produce an acquired immunity towards *Schistosoma*, which protects them to a certain degree [6]. It was found that the number of years performing the current job was positively correlated with the infection with *S. japonicum.* Our findings showed that the prevalence decreased significantly with increasing treatment times among this population. Prevalence was higher in the participant subgroup that did not receive any treatment in the past three years. These results all indicated that PZQ treatment is still an effective way to control schistosomiasis among this population, so as to promote the elimination of schistosomiasis in P. R. China [26].

One of the limitations of this study is that 152 fishermen and boatmen were excluded from the final analysis due to incomplete response on the questionnaire or lack of stool samples for PCR testing. This might have impacted on the study’s results. On the other hand, such a phenomenon reflects the difficulties of conducting control or prevention activities in low endemic areas after many years of intervention. To eliminate schistosomiasis, new approaches should be considered and explored towards improving the coverage rate of control activities in the population at risk and increasing their compliance to ensure the effectiveness of intervention activities.

## Conclusions

Our findings show that fishermen and boatmen remain the most vulnerable population for schistosome infection in endemic areas, and are an enormous obstacle in elimination of schistosomiasis in P. R. China. Among this population, younger fishermen and boatmen with longer occupational histories, as well as those with fewer treatments, particularly in the past three years, are at increased risk of acquiring infection. In addition, gaps exist in this population between knowledge, attitude and practices towards schistosomiasis prevention. Thus, it is suggested that enhanced medication, health promotion activities encouraging behavioral changes and other integrated approaches should be combined and strengthened to reduce the prevalence of schistosomiasis amongst this high-risk population.

## Supplementary information

**Additional file 1: Table S1.** Questionnaire of sociodemographic data and KAPs for fishermen and boatmen.

**Additional file 2: Table S2.** Univariate logistic regression analysis of variables associated with *S. japonicum* among the respondents.

## Data Availability

Data supporting conclusions of this article are included within the article and its additional files.

## Abbreviations

KAPs: knowledge, attitude and practices
PCR: polymerase chain reaction
NTDs: neglected tropical diseases
OR: odds ratio
95% CI: 95% confidence interval

## References

[1] WHO (2008). The social context of schistosomiasis and its control: an introduction and annotated bibliography.

[2] Steinmann P, Keiser J, Bos R, Tanner M, Utzinger J (2006). Schistosomiasis and water resources development: systematic review, meta-analysis, and estimates of people at risk. Lancet Infect Dis..

[3] Crompton DW (1999). How much human helminthiasis is there in the world?. J Parasitol..

[4] WHO. Schistosomiasis: progress report 2001–2011 and strategic plan 2012–2020. Geneva: World Health Organization; 2013. http://www.who.int/iris/bitstream/10665/78074/1/9789241503174_eng.pdf. Accessed 24 Apr 2020.

[5] Zhou XN, Wang LY, Chen MG, Wu XH, Jiang QW, Chen XY (2005). The public health significance and control of schistosomiasis in China—then and now. Acta Trop.

[6] Xu JF, Xu J, Li SZ, Jia TW, Huang XB, Zhang HM (2013). Transmission risks of schistosomiasis japonica: extraction from back-propagation artificial neural network and logistic regression model. PLoS Negl Trop Dis..

[7] Wang LD, Chen HG, Guo JG, Zeng XJ, Hong XL, Xiong JJ (2009). A strategy to control transmission of *Schistosoma japonicum* in China. N Engl J Med..

[8] He JC, Wang TP, Zhang SQ, Gao FH, Zhang GH, Yang WP (2011). Evaluation of mid-term effectiveness of medium and long term programme for prevention and control of schistosomiasis in Anhui Province. Chin J Schisto Control..

[9] Zeng XJ, Chen HG, Hong XL, Hu ZH, Jiang WS, Hu SZ (2012). Evaluation on medium-term effect of schistosomiasis comprehensive control strategy based on infectious source control in Poyang Lake area. Chin J Schisto Control..

[10] Zhou XN, Guo JG, Wu XH, Jiang QW, Zheng J, Dang H (2007). Epidemiology of schistosomiasis in the People’s Republic of China, 2004. Emerg Infect Dis..

[11] Zou L, Ruan S (2015). Schistosomiasis transmission and control in China. Acta Trop..

[12] Sun LP, Wang W, Zuo YP, Hong QB, Du GL, Ma YC (2017). A multidisciplinary, integrated approach for the elimination of schistosomiasis: a longitudinal study in a historically hyper-endemic region in the lower reaches of the Yangtze River, China from 2005 to 2014. Infect Dis Poverty..

[13] Colley DG, Bustinduy AL, Secor WE, King CH (2014). Human schistosomiasis. Lancet..

[14] Chitsulo L, Loverde P, Engels D (2004). Schistosomiasis. Nat Rev Microbiol..

[15] Abou-Zeid AHA, Abkar TA, Mohamed RO (2012). Schistosomiasis and soil-transmitted helminths among an adult population in a war affected area, Southern Kordofan state, Sudan. Parasit Vectors..

[16] Guan Z, Lu S, Li SZ, Dang H, Zhang LJ, Xu J (2018). Analysis on the situation of schistosome infections in floating population in national schistosomiasis surveillance sites of China. Chin J Schisto Control..

[17] Zhou XN, Bergquist R, Leonardo L, Yang GJ, Yang K, Sudomo M (2010). Schistosomiasis japonica: control and research needs. Adv Parasitol..

[18] Zhao GM, Zhao Q, Jiang QW, Chen XY, Wang LY, Yuan HC (2005). Surveillance for schistosomiasis japonica in China from 2000 to 2003. Acta Trop..

[19] Guan Z, Lu S, Li SZ, Xu J (2017). Endemic status of schistosomiasis in floating population and control challenges in P. R. China. Chin J Schisto Control..

[20] Xu J, Guan ZX, Zhao B, Wang YY, Cao Y, Zhang HQ (2015). DNA detection of *Schistosoma japonicum*: diagnostic validity of a LAMP assay for low-intensity infection and effects of chemotherapy in humans. PLoS Negl Trop Dis..

[21] Huang SY, Lin RX, Zhang QM, Deng ZH, Zhang XC, Huo LC (2010). Analysis of schistosomiasis surveillance in floating population in Guangdong, 2005–2009. South China J Prev Med..

[22] Odiere MR, Rawago FO, Ombok M, Secor WE, Karanja DM, Mwinzi PN (2012). High prevalence of schistosomiasis in Mbita and its adjacent islands of Lake Victoria, western Kenya. Parasit Vectors..

[23] Norton AJ, Gower CM, Lamberton PHL, Webster BL, Lwambo NJS (2010). Genetic consequences of mass human chemotherapy for *Schistosoma mansoni*: population structure pre- and post-praziquantel treatment in Tanzania. Am J Trop Med Hyg..

[24] Hotez PJ, Fenwick A (2009). Schistosomiasis in Africa: an emerging tragedy in our new global health decade. PLoS Negl Trop Dis..

[25] Yu XL, Zhou J, He YK, Huang MZ, Li YS (2013). Influence factors of *Schistosoma japonicum* infection among fishermen in eastern Dongting Lake Region. Chin J Parasitol Parasit Dis..

[26] Zhang J, Li ZJ, Qiu L, Li D, Chen JJ, Xie H (2016). Development and application of communication materials for participatory health education of schistosomiasis in fishermen and boatmen of Poyang Lake region. Chin J Schisto Control..

[27] Zhou J, Huang CY, He YK, Du YQ, Yu XL, Wang YY (2010). Epidemiological evaluation of schistosomiasis in migrant fishermen in Dongting Lake region. Chin J Schisto Control..

[28] Cao CL, Bao ZP, Zhu HQ, Yu Q, Li SZ, Wang Q (2010). Analysis of schistosomiasis control requirements in boat fishermen in lake regions. Chin J Schisto Control..

[29] Chen JH, Wen LY, Zhang XZ, Zhang JF, Yu LL, Hong LD (2006). Development of a PCR assay for detecting *Schistosoma japonicum*-infected *Oncomelania hupensis*. Chin J Parasitol Parasit Dis..

[30] Wang S, Carlton EJ, Chen L, Liu Y, Spear RC (2013). Evaluation of an educational intervention on villagers’ knowledge, attitude and behaviour regarding transmission of *Schistosoma japonicum* in Sichuan province, China. Acta Trop..

[31] Zhou LY, Deng Y, Steinmann P, Yang K (2013). The effects of health education on schistosomiasis japonica prevalence and relevant knowledge in the People’s Republic of China: A systematic review and meta-analysis. Parasitol Int..

[32] Hu GH, Jia H, Song KY, Lin DD, Zhang J, Cao CL (2005). The role of health education and health promotion in the control of schistosomiasis: experiences from a 12-year intervention study in the Poyang Lake area. Acta Trop..

[33] Cawston FG (2010). Schistosomiasis in southern Africa. Acta Med Scand..

[34] Zhou XN, Xu J, Chen HG, Wang TP, Huang XB, Lin DD (2011). Tools to support policy decisions related to treatment strategies and surveillance of schistosomiasis japonica towards elimination. PLoS Negl Trop Dis..

[35] Zhang LJ, Xu ZM, Qian YJ, Dang H, Lu S, Xu J (2016). Endemic status of schistosomiasis in People’s Republic of China in 2015. Chin J Schisto Control..

[36] Yi DH, Yi P, Liu ZC, Li YS, Quan MZ, Xiao SY (2009). Practice and thought of schistosomiasis control with an emphasis on control sources of infection in Dongting Lake area. Chin J Schisto Control..

[37] Pontes LA, Diasneto E, Rabello A (2002). Detection by polymerase chain reaction of *Schistosoma mansoni* DNA in human serum and feces. Am J Trop Med Hyg..

[38] Malenganisho WLM, Magnussen P, Friis H, Siza J, Vennervald BJ (2008). *Schistosoma mansoni* morbidity among adults in two villages along Lake Victoria shores in Mwanza District, Tanzania. Trans R Soc Trop Med Hyg..

[39] Ni L, Li PY, Mao XZ (2010). Four predicaments facing Dongting Lake fishermen got ashore. J Hunan Agric Univ..

[40] Fabri RL, Florêncio JR, Pinto NDCC, Mattos ACA, Coelho PMZ, Vasconcelos EG (2014). Chromatographic fingerprint analysis and effects of the medicinal plant species *Mitracarpus frigidus* on adult *Schistosoma mansoni* worms. Biomed Res Int..

