# Supplementary material for: Assessment of knowledge, attitude and practices and the analysis of risk factors regarding schistosomiasis among fishermen and boatmen in the Dongting Lake Basin, the People’s Republic of China
Source: Parasit Vectors. 2020 Jun 1;13:273. doi: 10.1186/s13071-020-04157-4 (PMC7268453; doi:10.1186/s13071-020-04157-4)
Supplement: Supplementary file 1 — Additional file 1: Table S1. Questionnaire of sociodemographic data and KAPs for fishermen and boatmen. [file 13071_2020_4157_MOESM1_ESM.docx]

**Additional file 1: Table S1. Questionnaire of sociodemographic data and KAPs for fishermen and boatmen**

ID code□□□□□□□

**1.Sociodemographic data**

Name

Gender：□ 1.Male 2.Female Date of Birth： year month day

Occupation： 口1．Professional fisherman 2．Professional boatman

3．Sideline fisherman and boatman

Living condition：口1．Living on boat permanently 2．Living on boat only during fishing season 3．Never living on boat

Education：□ 1. Below primary school 2. Primary school 3. Junior middle school

4. Senior middle school 5. Above high school

Annual income：□ 1. Less than 5000 2. 5000-10000 3. 10000-20000 4. More than 20000

Years of doing current job： year

Whether contacted infected water in last one year □ 1. Yes 2. No 9. Don’t know

If yes，in which month did you contact ( There may be more than one answer )

Major site exposed to endemic water：□ 1. Dongting Lake 2. Tributaries of Dongting Lake

3. Yangtze River 4. Other waters

Whether had symptoms in the last two months：□ 0. No 1. Yes

Infection history：□ 0. No 1. Yes

If Yes，answer the following two questions

Last diagnosis method：□ 1. Ask 2. Serological detection 3. Stool examination 9. Don’t know

Last diagnosis category: □ 1. Acute 2. Chronical 3. Advanced 9. Don’t know

Times received treatment(s)：

Whether received treatment in recent two years： □ 1. Yes 2. No 9. Don’t know

**2. Knowledge on schistosomiasis control**

(1) Transmission season of schistosomiasis is mainly in [ ]

(A) January to February (B) March to April

(C) April to October (D) November to December

(2) Susceptible population of schistosomiasis is [ ]

(A) Adult (B) Children (C) Everyone (D) Don’t know

(3) Which of the following animals cannot be infected with *S. japonicum*? [ ]

(A) Cattle (B) Sheep (C) Dog (D) Duck

(4) People get infected with *S. japonicum* because [ ]

(A) Breathed stuffy air (B) Shook hands with patients

(C) Contacted infected water (D) Ate stale food

(5) Intermediate host of schistosome is [ ]

(A) Field snail (B) Oncomelania (C) Shrimp (D) Frog

(6) Main symptoms of schistosomiasis are [ ]

(A) Heartbeat quickening (B) Fever and diarrhea

(C) Hand and foot disorder (D) Don’t know

(7) Effect of schistosomiasis on children may be [ ]

(A) Self-recovering (B) Physical growth Limitation (C) Don’t know

(8) Effect of schistosomiasis on women is [ ]

(A) Self- recovering (B) Severe impacts (C) Don’t know

(9) Drug against schistosomiasis is [ ]

(A) Praziquantel (B) Toluidazole (C) Niclosamide (D) Don’t know

(10) Most direct way to prevent schistosomiasis is [ ]

(A) Don’t contact with patients (B) Don’t contact with feces

(C) Don’t contact with infected water

**3. Attitude of schistosomiasis prevention and control**

(1) Are you willing to install feces container on your boat? [ ]

(A) Yes (B) No

If No，Why?（There may be more than one answer） [ ]

(A) Troublesome (B) Boat is too small to install

(C) Feces tend to spill over (D) Smell bad

(2) Are you willing to use onshore toilets? [ ]

(A) Yes (B) No

If No，Why?（There may be more than one answer） [ ]

(A) It’s difficult to use onshore toilets when the ships are far away from the shore.

(B) It’s inconvenient to use onshore toilets when the ships pulled over.

(3) Are you willing to take examination of schistosomiasis when doctors come? [ ]

(A) Go for it directly (B) Go only when summoned (C) Reject

If Reject，Why?（There may be more than one answer）： [ ]

(A) Too troublesome (B) There is no need to examine without feeling uncomfortable

(C) Taking medicine directly is enough (D) Other reasons

(4) If diagnosed, are you willing to take medicine regularly? [ ]

(A) Yes (B) No

If No，Why?（There may be more than one answer） [ ]

(A) Don’t believe that taking medicine can cure schistosomiasis

(B) There is no need to take medicine without feeling uncomfortable

(C) Always get re-infected after treatment

(D) Worry about reactions of drug

(E) Other reasons

(5) Can schistosomiasis be prevented? [ ]

(A) Yes (B) No

(6) Can schistosomiasis be cured? [ ]

(A) Yes (B) No

**4. Practices towards schistosomiasis control**

(1) Have you installed feces container on your boat and did you use it? [ ]

(A) Didn’t installed feces container

(B) Have already installed and used, then poured it in toilets onshore

(C) Have already installed and used, then poured it into the lake

(D) Have already installed but didn’t use it

(2) Where do you usually defecate? [ ]

(A) Defecate in lake or river (B) Defecate in onshore toilets (C) Defecate at home

(3) Do you protect yourself by wearing cloths & gloves or taking medicine when contacting freshwater? [ ]

(A) Always (B) Sometimes (C) Never

If Sometimes or Never, Why (There may be more than one answer)： [ ]

(A) There’s no need (B) Too troublesome

(C) Can't protect hands when wearing rubber shoes or rubber clothes.

(D) Too hot to wear rubber shoes or rubber clothes during hot days
