# Supplementary material for: Assessment of knowledge, attitude and practices and the analysis of risk factors regarding schistosomiasis among fishermen and boatmen in the Dongting Lake Basin, the People’s Republic of China
Source: Parasit Vectors. 2020 Jun 1;13:273. doi: 10.1186/s13071-020-04157-4 (PMC7268453; doi:10.1186/s13071-020-04157-4)
Supplement: Supplementary file 2 — Additional file 2: Table S2. Univariate logistic regression analysis of variables associated with S. japonicum among the respondents. [file 13071_2020_4157_MOESM2_ESM.docx]

**Additional file 2: Table S2. Univariate logistic regression analysis of variables associated with *S. japonicum* among the respondents**

| **Risk factors** | **Positive rate determined by PCR**  **(No. positives/No. examined)** | **OR (95%CI)** | **P-value** |
| --- | --- | --- | --- |
| **Gender** | | | |
| male | 14.78% (51/345) | 1 |  |
| female | 12.5% (32/356) | 0.824 (0.512-1.324) | 0.4230 |
| **Age group (years)** | | | |
| <30 | 38.10% (8/21) | 1 |  |
| 30- | 27.78% (15/54) | 0.625 (0.216-1.81) | 0.3862 |
| 40- | 15.35% (35/228) | 0.295 (0.114-0.763) | 0.0118 |
| 50- | 10.33% (19/203) | 0.187 (0.069-0.509) | 0.0010 |
| ≥60 | 5.26% (6/114) | 0.09 (0.027-0.301) | <.0001 |
| **Occupation** | | | |
| Professional boatman | 27.27% (3/11) | 1 |  |
| Professional fisherman | 18.70% (43/230) | 0.613 (0.156-2.407) | 0.4833 |
| Part-time fisherman or boatman | 10.28% (37/360) | 0.305 (0.078-1.202) | 0.0897 |
| **Living situation** | | | |
| Living on board all year round | 11.76% (2/17) | 1 |  |
| Living on board only in fishing season | 11.81 (15/127) | 1.004 (0.209-4.831) | 0.9956 |
| Never living on board | 14.44% (66/457) | 1.266 (0.283-5.664) | 0.7577 |
| **Education** | | | |
| Illiteracy or self-illiteracy | 19.30% (11/57) | 1 |  |
| Primary school | 12.57% (5/57) | 0.601 (0.27-1.34) | 0.2137 |
| Junior middle school | 12.96% (13/324) | 0.623 (0.299-1.297) | 0.2057 |
| High school and higher education | 16.98% (3/53) | 0.855 (0.323-2.263) | 0.7530 |
| **Family income (Chinese Yuan)** | | | |
| <5000 | 11.24% (19/169) | 1 |  |
| 5000- | 18.56% (49/264) | 1.799 (1.018-3.179) | 0.0431 |
| 10000- | 9.41% (8/85) | 0.82 (0.343-1.959) | 0.6555 |
| ≥20000 | 8.43% (7/83) | 0.727 (0.293-1.806) | 0.4923 |
| **Years of doing current job** | | | |
| <10 | 7.61% (7/92) | 1 |  |
| 10- | 12.86% (49/381) | 1.792 (0.784-4.098) | 0.1668 |
| ≥30 | 21.09% (27/128) | 3.246 (1.346-7.825) | 0.0087 |
| **Contact with the infected water last year** | | | |
| No | 10.09% (11/109) | 1 |  |
| Yes | 14.63% (72/492) | 1.527 (0.78-2.989) | 0.2164 |
| **Symptom** | | | |
| No | 12.76% (61/478) | 1 |  |
| Yes | 17.89% (22/123) | 1.489 (0.873-2.539) | 0.1437 |
| **Infection history** | | | |
| No | 4.92% (3/61) | 1 |  |
| Yes | 14.81% (80/640) | 3.362 (1.029-10.992) | 0.0448 |
| **Treatment times** | | | |
| 0 | 32.00% (8/25) | 1 |  |
| 1- | 17.13% (37/216) | 0.439 (0.176-1.093) | 0.0770 |
| 6- | 12.26% (26/212) | 0.297 (0.117-0.757) | 0.0110 |
| ≥10 | 8.11% (12/148) | 0.187 (0.067-0.524) | 0.0014 |
| **Treatment in 2015-2017** | | | |
| No | 18.18 % (40/220) | 1 |  |
| Yes | 11.29% (43/381) | 0.572 (0.359-0.913) | 0.0192 |

OR, odds ratio. CI, Confidence interval.*Significant association (P<0.05)
